# Human T Lymphotropic Virus and Pulmonary Diseases

**DOI:** 10.3389/fmicb.2018.01879

**Published:** 2018-08-14

**Authors:** Apio R. N. Dias, Luiz F. M. Falcão, Aline S. C. Falcão, Valéria M. F. Normando, Juarez A. S. Quaresma

**Affiliations:** ^1^Tropical Medicine Center, Federal University of Pará, Belém, Brazil; ^2^Center of Health and Biological Sciences, State University of Pará, Belém, Brazil; ^3^Graduate Program of Virology, Evandro Chagas Institute, Ministry of Health, Ananindeua, Brazil

**Keywords:** HTLV-1, HAM/TSP, ATL/L, pulmonary disease, pulmonary function

## Abstract

Human T lymphotropic virus type 1 (HTLV-1) is the etiological agent of HTLV-1-associated myelopathy, and adult T cell lymphoma/leukemia (ATL/L). Pulmonary complications such as alveolitis and bronchiectasis were found in individuals who develop TSP/HAM due to chronic inflammation. These individuals showed image anomalies in CT scans and changes in pulmonary function parameters distinctive of pulmonary disease. Furthermore, infected individuals have a greater susceptibility to pulmonary tuberculosis either due to changes in the innate immune response, in asymptomatic carriers, or to an opportunistic disease linked to immunodepression, in individuals who develop ATL/L. This summary addresses the general lack of knowledge regarding the relationship between HTLV-1 and pulmonary diseases and provides direction for future work.

## Introduction

Human lymphotropic virus type 1 (HTLV-1) is the etiological agent of HTLV-1-associated myelopathy, and adult T-cell lymphoma/leukemia (ATL/L) (Richardson et al., 1990). Various HTLV related pulmonary anomalies heavily rely on the clinical form manifested in the infected individual. In ATL/L, the pneumophathies mainly are caused by opportunistic infections or pulmonary leukemic infiltrates. In asymptomatic carriers and HAM/TSP patients, T lymphocytic alveolitis, interstitial pneumonia, bronchiolitis, and diffuse panbronchiolitis could be observed. Besides, an elevated risk of developing pulmonary cryptococcosis, tuberculosis, and community acquired pneumonia was observed (Atsumi et al., 2009; Fukuoka et al., 2013).

A relationship between HTLV-1 and pulmonary diseases was established with reports of individuals with TSP/HAM who exhibit pulmonary diseases with characteristics of lymphocytic inflammatory infiltrate (Sugimoto et al., 1987) and later by other authors (Teruya et al., 2008; Kawabata et al., 2012; Nakayama et al., 2013), include opportunistic pulmonary infections (Taylor and Matsuoka, 2005) and alterations in CT images in infected individuals (Kikuchi et al., 1996; Tateishi et al., 2001; Kadota et al., 2004; Okada et al., 2016; Einsiedel et al., 2014; Yamakawa et al., 2015; Falcão et al., 2017).

Despite several studies linked HTLV-1 to pulmonary diseases, the scientific community still has several questions to solve about the physiopathology of HTLV-1 infection and understand why do some HTLV-1-infected individuals have pulmonary damage while others do not. A recent study in Australia related highest mortality rates among subjects infected with HTLV-1c subtype (Einsiedel et al., 2018). Another question remains unclear, for example, the role of epigenetic factors or inflammatory imbalance in the lung injury.

This study aims to improve the understanding of lung disease in patients infected with HTLV, through a review of publications on this neglected theme, gathering information from studies on the area including recent reports.

## Immune Response

Patients with HTLV-1 with pulmonary involvement exhibit an elevation of T lymphocytes in bronchoalveolar lavage fluid (BALF) and consequent pulmonary inflammation, which is mediated by a Th1 type immune response, characterized by the presence of soluble IL-2 receptors (IL-2R), increased levels of interleukins IL-2, IL-12, and interferon (IFN-γ) (Sugimoto et al., 1989; Yamazato et al., 2003; Nakayama et al., 2013). An increase in the expression of IL-10, an anti-inflammatory cytokine, has also been detected. Its production is related to a reduction in the activation of T cells responsible for the chronic inflammatory process (Nakayama et al., 2013). The mechanism underlying pulmonary inflammation involves the local release of cytokines and chemokines and is related to the interaction between the HTLV-1 bZIP factor (HBZ) and forkhead box P3 (Foxp3) in infected CD4+ T cells (Nakayama et al., 2013).

**FIGURE 1:**
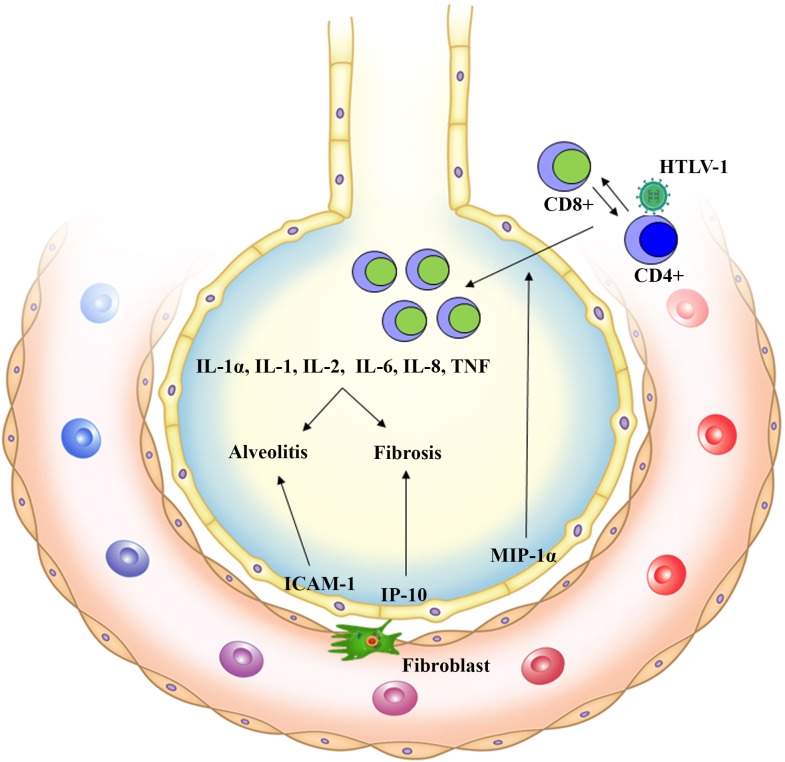
Pulmonary inflammation is induced by HTLV-1 cells infected by HTLV-1 (T CD4+, T CD25+, and Lung Epithelial cells). MIP-1α chemokine recruits and activates inflammatory cells to the lungs (HTLV-1 specific T CD8+ cells) expressing inflammatory cytokines (IL-1α, IL-1, IL-2, IL-6, IL-8, and TNF). There is an increase in IP-10, a chemokine that plays a key role in the pathogenesis of pulmonary fibrosis. Associated with this, ICAM-1 acts by facilitating neutrophil adhesion to pulmonary epithelial cells and potentiating local chronic inflammation. The result is the development of Alveolitis and Fibrosis.

Another characteristic of pulmonary disorders in patients with HTLV-1 is the presence of alveolitis (cryptogenic fibrosing alveolitis) and lymphocytosis (CD4+ and CD25+) (Matsuyama et al., 2003), in another study that evaluates the peripheral blood monocyte cells (PBMCs) and BALF from six patients (five HAM/TSP and one HTLV-1 carrier) who had pulmonary involvement, lymphocytosis and the presence of HTLV-1 provirus in the BALF of HTLV-1 patients was found (Kawabata et al., 2012). These findings are positively correlated with elevated levels of MIP-1α, a chemokine responsible for the activation and recruitment of inflammatory cells, IP-10, which plays a key role in the pathogenesis of pulmonary fibrosis, and ICAM-1. Various cells of the pulmonary epithelium express ICAM-1, which facilitates the adhesion of neutrophils to cells of the respiratory epithelium and thereby potentiates chronic inflammation which is the landmark of many diseases related to HTLV-1 (Miyazato et al., 2000; Matsuyama et al., 2003) (**Figure 1**).

A local production of ICAM-1, cytokines (IL-1α, IL-1, IL-6, IL-8, and TNF), and chemokines (CCL2 and CCL5) is believed to be induced by the NF-κβ and AP-1 pathways in HTLV-1-infected epithelial cells; this mechanism can explain, in part, the pathogenesis of HTLV-1-related lung diseases characterized by the production of inflammatory cytokines and chemokines, and expression of adhesion molecules by the infected cells (Teruya et al., 2008). Patients with TSP/HAM and uveitis as well as asymptomatic carriers may present pulmonary complications characterized by T-lymphocytic alveolitis. In previous studies based on BALF analyses, the proportion of lymphocytes at the pulmonary level was higher in HTLV-1 infected individuals than in individuals with pulmonary changes and negative serology for HTLV-1 (Sugimoto et al., 1987; Mattos et al., 1995).

The main pulmonary lesions found in patients with HTLV-1 are alveolitis and bronchiectasis resulting from the increase in inflammatory chemokines (MIP-1α and IP-10) induced by Tax, which is responsible for the activation and circulation of inflammatory cells and their recruitment to the lungs; and by ICAM, which promotes leukocyte adhesion to the pulmonary endothelium (Matsuyama et al., 2003). Other findings include increased mRNA expression of the viral *Tax* and *HBZ* in BALF cells of patients with HTLV-1. Additionally, there is a positive correlation between high levels of *Foxp3* mRNA and a high proportion of lymphocytes in BALF of patients with HTLV-1-related lung diseases. These findings suggest the involvement of regulatory T cells in the pathogenesis of pulmonary inflammation (Nakayama et al., 2013).

There are findings showing that HTLV-1-specific CD8+ T cells accumulate in the BALF at a higher concentration than that in the peripheral blood of patients infected with HTLV-1, suggesting that selective infiltration occurs in the lungs and specific immune responses occur in lung tissues (Mori et al., 2005; Kawabata et al., 2012). This hypothesis is supported by a histochemical analysis revealing the formation of clusters of CD8+ T cells in the lung tissue of a patient with TSP/HAM (Kawabata et al., 2012).

**FIGURE 2:**
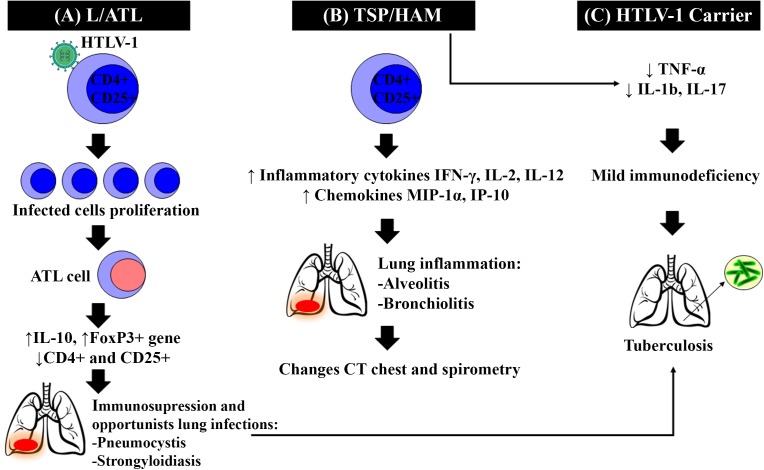
HTLV-1 clinical forms and their pulmonary alterations. After HTLV-1 infection, three clinical forms may occur. **(A)** ATL/L Clonal proliferation of infected cells, through the actions of Tax and others viral proteins, causes alterations and errors in the host genome, leading to the onset of ATL cells, reduced numbers of naive T cells and T reg cells (CD4+ and CD25+). ATL/L also induces higher expression of Forkhead Fox P3+ and anti-inflammatory cytokines, such as IL-10, leading to immunodeficiency and opportunistic infections as Tuberculosis. **(B)** HAM/TSP The increased levels of lymphocytes (CD4+ and CD25+), inflammatory cytokines as Interleukine 2 (IL-2), Interleukine 12 (IL-12), and Interferon-γ (IFN-γ), and inflammatory chemokines such as Macrophage inflammatory protein (MIP-1α) and IP-10 in the BALF, leading to the development of alveolitis and bronchiolitis. **(C)** Asymptomatic Carriers do not develop visible symptoms but exhibit tumor necrosis factor-α (TNF-α), Interleukine 1-β (IL-1β) and Interleukine 17 (IL-17) low expression, which makes these individuals more susceptible to coinfection by *Mycobacterium tuberculosis*, developing more severe forms of the disease.

Patients with HTLV-1-associated inflammatory diseases (e.g., HAM/TSP and uveitis) exhibit more substantial lung tissue injury, as evidenced by the significantly increased levels of lymphocytes (CD4+ and CD25+), cytokines (IL-2, IL-12, and IFN-γ), and inflammatory chemokines (MIP-1α and IP-10) as well as cell adhesion molecules (ICAM-1) in the BALF compared to the levels observed in asymptomatic carriers (Yamazato et al., 2003; Nakayama et al., 2011, 2013). These findings support the relationship between the occurrence of diseases related to HTLV-1 and the development of alveolitis (Matsuyama et al., 2003) (**Figure 2B**).

The virus subtype seems to be correlated with the severity of the disease. In Australian indigenous people, the highest mortality rate was found among subjects infected with HTLV-1c subtype and high proviral load (pVL > 1000 copies per 10^5^ peripheral blood leukocytes) when compared to those infected with the same virus but with lower proviral load or uninfected subjects. This mortality was largely due to bronchiectasis-related deaths (Einsiedel et al., 2018). It is believed that the link between HTLV-1 associated diseases and higher proviral load result from a genetically determined, inefficient cytotoxic T lymphocyte response, which permit the widespread dissemination of the virus (Saito and Bangham, 2012).

The HTLV-1 proviral load in the BALF seems to be related with the proportion of lymphocytes in the BALF too (Desgranges et al., 1989). Besides that, the higher percentages of CD4+ and CD25+ in the BALF than in peripheral blood (Seki et al., 2000) suggests that HTLV-1 infection could induce chronic inflammation in the lung, through immunologic mechanisms (Yamakawa et al., 2015). It seems that this exacerbated immune response that occurs in HTLV-1 infected patients may play a key role in the development of lung injury.

## HTLV-1/Tuberculosis Coinfection and ATL/L

The high prevalence of HTLV-1 among patients with pulmonary tuberculosis (PT) (Moreira et al., 1993; Pedral-Sampaio et al., 1997; Marinho et al., 2005; Verdonck et al., 2008; Bastos et al., 2009; Kozlowski et al., 2014), high mortality rates among co-infected individuals, and increased likelihood of hospitalization and treatment for PT in patients with HTLV-1 (Bastos et al., 2012) reinforce the hypothesis that individuals with HTLV-1 are at higher risk of infection with *Mycobacterium tuberculosis* (Marinho et al., 2005; Grassi et al., 2016).

This was also demonstrated in the Northeast Region of Brazil (Moreira et al., 1993; Pedral-Sampaio et al., 1997; Marinho et al., 2005; Bastos et al., 2009) and in a study conducted in the Central-west region of Brazil, where the prevalence was higher than that observed in blood donors (Kozlowski et al., 2014). In a study conducted with patients admitted to a hospital in Salvador, Brazil, 2 deaths were observed in HTLV-1 and tuberculosis coinfected patients (Bastos et al., 2012).

A cohort study conducted in Salvador, Bahia, Brazil, found a higher incidence density of 3,3 per 1,000 people in an HTLV-1 infected group. The higher risk of PT was found mainly in individuals aged 31 to 50 years (Grassi et al., 2016). In another study conducted in Lima, Peru the authors found an association between HTLV-1 infection and a lifetime history of active PT among relatives of HTLV-1 infected patients (Verdonck et al., 2008). These findings reinforce the concepts of an association between HTLV-1 and PT in areas where both infections are endemic, and the increased risk of HTLV-1 individuals to develop PT.

The increased susceptibility to infection appears to be linked to a change in the innate immune response associated with HTLV-1, which facilitates the multiplication of the etiological agent of PT and leads to a more severe disease (Marinho et al., 2005). HTLV-1 infection increases the risk for tuberculosis, by the way, little is known. It is probable that HTLV-1 leads to decreased production of TNF-α, which plays a key role in protection against *M. tuberculosis* (Bastos et al., 2012; Carvalho et al., 2018). Furthermore, the production of interleukins (IL-1b and IL-17) was lower in HTLV-1 infected and co-infected individuals when compared with healthy subjects. This impairment in TNF, IL-1b and IL-17 production upon stimulation with mycobacterial antigens, may explain the higher susceptibility to *M. tuberculosis* in HTLV-1 infected individuals (Carvalho et al., 2018). The exaggerated inflammation in HTLV-1 infection may have contributed to the rapid sputum smear conversion and severity of tuberculosis (**Figure 2C**).

In individuals with ATL/L, HTLV-1 infection causes a clonal proliferation of infected cells by action Tax and others viral proteins. Alterations and errors in the host genome accumulation leading to the onset of ATL cells with reduction in the number of naive T cells, T reg cells (CD4+ and CD25+) express of FoxP3+ phenotype anti-inflammatory cytokines, such as IL-10, suggesting an immunoregulatory function leading to immunodeficiency (Taylor and Matsuoka, 2005) (**Figure 2A**). It causes more susceptibility to this individuals develop opportunistic infections by several pathogens (Taylor and Matsuoka, 2005). In a 3712 ATL/L patients cohort of a Japanese hospital, pulmonary tuberculosis had a prevalence of 3.8% that were significantly associated with higher health care costs (Maeda et al., 2015).

## CT Findings and Pulmonary Function

Computed tomography (CT) abnormalities in patients with HTLV-1 are predominantly parenchymal, especially centrilobular nodules, thickening of bronchovascular bundles, ground-glass opacity, and bronchiectasis. There is an association between HTLV-1 infection and bronchiectasis (Honarbakhsh and Taylor, 2015). In a case report, two HTLV-1 carriers were diagnosed with interstitial pneumonia, both showed pulmonary alterations in CT but low lymphocytic infiltration (Burioka et al., 1997). In a retrospective case review of 246 asymptomatic and 167 symptomatic patients, 13 symptomatic patients was diagnosed with bronchiectasis (13/167, RR 19,2 95% CI 2,5 – 14,5, *p* = 0.004) (Honarbakhsh and Taylor, 2015), and this association is particularly strong in patients with inflammatory diseases associated with the virus (e.g., uveitis and HAM/TSP) living in regions with low socio-economic development (Einsiedel et al., 2014). In a study with 645 patients, bronchiectasis was the most frequently observed abnormality (51.3%, *n* = 19) (Nakayama et al., 2013).

In patients with HAM/TSP, combinations of alterations are frequently found in CT, including bronchiectasis and pleural thickening, bronchiectasis and parenchymal bands, bronchiectasis and interlobular septum thickening, bronchiectasis and centrilobular nodules, and centrilobular nodules and parenchymal bands, with some patients presenting three or more changes (Falcão et al., 2017). Other imaging findings that reinforce the existence of a causal relationship between pulmonary diseases and HTLV-1, including findings of peripheral bronchioles and alveoli at sites of injury, probably due to BALF lymphocytosis (Kawabata et al., 2012) as well as a higher prevalence of pneumonias among patients with HTLV-1 than that in the general population (Yamashiro et al., 2012).

A study involving indigenous individuals in Australia (840 patients) shows higher rates of CT with bronchiectasis between the individuals infected with high proviral loads. All individuals were infected with HTLV-1c, and although HTLV-1 associated bronchiectasis affects diverse genetic backgrounds, the infected individuals in Japan use to have subclinical lung disorders. It was demonstrated that the HTLV-1 infected indigenous adults have more diffuse bronchiectasis and a higher HTLV-1c proviral load, which correlates with more extensive injuries and deaths due to bronchiectasis. The authors demonstrate that rates of bronchiectasis and bronchitis/bronchiolitis were significantly higher in this population than among the healthy individuals (adjusted odds ratio = 2.9; 95% CI, 2.0, 4.3) they propose that this viral subtype is linked with major lung injury lesions between this population (Einsiedel et al., 2018).

Also, certain studies that try to correlate the CT image findings with alterations in the respiratory function. In a study with asymptomatic individuals, no difference in parameters of respiratory function was found when compared to those of a healthy control group. These results suggest that the HTLV associated pulmonary abnormalities previous described in CT images, cells, and BALF could not affect the pulmonary function, but in this study, the parameters were not evaluated in symptomatic individuals (Murphy et al., 2003).

We show that may be a relationship between HTLV-1 infection and changes in pulmonary function, especially in individuals with HAM/TSP, which may progress to obstructive or restrictive lung disease, possibly related to pulmonary inflammation (Normando et al., 2016; Falcão et al., 2017). A previous study observed obstructive disease in a suspect patient of bronchiolitis obliterans that was identified as HTLV-1 associated bronchioloalveolar disorder (HABA) with 1.25 l forced expiration volume in one second (FEV_1_) (74.4% of predicted) and 64.1% forced expiratory volume in one second/forced vital capacity (FEV_1_/FVC) (Yamakawa et al., 2015) due Adult T-cell leukemia (Kimura, 1992).

Spirometry analysis in patients with HAM/TSP has shown a reduction in vital capacity (VC), FEV_1_, and maximum voluntary ventilation (MVV). Low VC is related to restrictive lung disease, a reduction of FEV_1_ is correlated with airway obstruction, and a reduction of MVV is related to both conditions and to reduced mobility. Therefore, it is possible that the lesions found in imaging studies associated with reduced mobility affecting patients with HAM/TSP play a key role in the development of obstructive or restrictive lung diseases (Falcão et al., 2017). It is possible that bronchiectasis and pleural thickening play key roles in the development of obstructive disease and restrictive lung disease, respectively.

As a diagnostic criterion for the prevalence of HTLV-1 infection in patients with diffuse panbronchiolitis (Yamamoto et al., 2004) and cryptogenic fibrosing alveolitis (Matsuyama et al., 2003), spirometry shows a significant reduction of FEV_1_ in seropositive to seronegative patients. The reduction in the number of pulmonary CD4+ T lymphocytes reduces the ability to generate specific antigen responses, resulting in immunosuppression and therefore explaining the vulnerability to infections even in the presence of immunologic effectors. The early complement diagnostic of pulmonary function could inform an adequate intervention to avoid possible complications (Matsuyama et al., 2003).

## Future Directions

Pulmonary changes caused by HTLV-1 appear to be related to the release of cytokines (IL-1α, IL-1β, IL-6, IL-8, and TNF-α) and chemokines (MIP-1α and IP-10), which damage lung tissues via the infiltration of activated T CD8 + lymphocytes with an altered immune response pattern due to the expression of Tax and HBZ. HTLV-1 causes persistent inflammation in all organs in which it manifests. In the lungs, the characteristic fibrosis of these lesions is related to chronic inflammation, leading to the activation of pulmonary fibroblasts.

The HTLV-1 virus appears to have a peculiar tropism in relation to the lungs that is expressed by the pulmonary lesions found in individuals with HAM/TSP. In most cases, pulmonary symptomatology in patients with HTLV-1 is subclinical; however, further investigations are necessary to determine the evolution of the pathology in individuals with advanced pulmonary disease. The pulmonary inflammatory process related to HTLV-1, when associated with other inflammatory characteristics, may lead to a worse prognosis. Individuals infected with more aggressive viral subtypes and high proviral loads shows more severe lung injury, e.g., cases of Australian indigenous infected with HTLV-1c, ATL/L patients showed lung injury due to opportunistic infections, as Tuberculosis and Pneumonia caused by HTLV-1 infection induced immunodeficiency. On the other hand, the asymptomatic carriers seem to suffer from milder immunodeficiency due to the development of coinfection with *M. tuberculosis*.

The findings of lung lesions are more prominent in patients with some degree of clinical presentation (HAM/TSP), suggesting that lesions at the pulmonary level follow lesions in other organs, as well as the systemic inflammatory process. Accordingly, it may be appropriate to consider HTLV-1 infection as a systemic inflammatory disease of chronic evolution. Pulmonary lesions in asymptomatic individuals appear to be explained by an inaccurate classification, including in this category individuals with only one symptom. To better determine the course of the infection, well-defined classification criteria should be used. We believe that the most appropriate classification is the De Castro-Costa et al. (2006) criteria, in which individuals that already present some clinical manifestation are classified as definitive, probable, or possible HAM/TSP; the use of this classification in future studies would allow more effective comparisons.

Many studies made until now still do not allow a comprehensive understanding of pathophysiological mechanisms and their links to specific clinical presentations of patients infected by HTLV-1. There are mainly observational studies, who did not characterize the various mechanisms involved in lung alterations associated with HTLV-1 infection, these studies have limited scope or describe only isolated clinical cases. They do not answer the question about the evolution and physiopathology of the HTLV-1 related pulmonary disease, which remains unclear. Prospective studies and case-control studies are required to understand these mechanisms better, properly characterize the pulmonary involvement associated with HTLV-1 infection and to design strategies for disease treatment or management. This review intends to contribute with this reflections and provides direction for the future investigations.

## Author Contributions

LF, AF, JQ, and AD conceptualized and designed the study, contributed to data acquisition, data interpretation, drafting of the manuscript, final approval of the version to be published, and drawing the figures. LF, AF, VN, AD, and JQ contributed to data interpretation, drafting of the manuscript, provided study supervision, contributed to data interpretation, and provided critical revision of the manuscript for important intellectual content. LF and AD contributed to data acquisition or analysis and editing of the manuscript for intellectual content.

## Conflict of Interest Statement

The authors declare that the research was conducted in the absence of any commercial or financial relationships that could be construed as a potential conflict of interest. The reviewer DG and handling Editor declared their shared affiliation at time of review.

## References

[B1] Atsumi E. Yara S. Higa F. Hirata T. Haranaga S. Tateyama M. (2009). Influence of human T lymphotropic vírus type I infection on the etiology of community-acquired pneumonia. *Intern. Med.* 48 959–965. 10.2169/internalmedicine.48.1918 19525581

[B2] Bastos M. L. Osterbauer B. Mesquita D. L. Carrera C. A. Albuquerque M. J. Silva L. (2009). Prevalence of HTLV-1 infection in hospitalized patients with tuberculosis. *Int. J. Tuberc. Lung Dis.* 13 1519–1523. 19919770 PMC2963180

[B3] Bastos M. L. Santos S. B. Souza A. Finkmoore B. Bispo O. Barreto T. (2012). Influence of HTLV-1 on the clinical, microbiologic and immunologic presentation of tuberculosis. *BMC Infect. Dis.* 12:199. 10.1186/1471-2334-12-199 22925731 PMC3449207

[B4] Burioka N. Suyama H. Sugimoto Y. Chikumi H. Yajima H. Tomita K. (1997). Intersticial pneumonia developed in HTLV-1 carriers: report of two cases. *Yonago Acta Med.* 40 125–131.

[B5] Carvalho N. B. Bastos M. L. Souza A. S. Netto E. M. Arruda S. Santos S. B. (2018). Impaired TNF, IL-1β, and IL-17 production and increased susceptiblity to *Mycobacterium tuberculosis* infection in HTLV-1 infected individuals. *Tuberculosis* 108 35–40. 10.1016/j.tube.2017.10.004 29523325

[B6] De Castro-Costa C. M. Araújo A. Q. Barreto M. M. Takayanagui O. M. Sohler M. P. da Silva E. L. (2006) Proposal for diagnostic criteria of tropical spastic paraparesis/HTLV-I-associated myelopathy (TSP/HAM). *AIDS Res. Hum. Retroviruses* 22 931–935. 10.1089/aid.2006.22.931 17067261

[B7] Desgranges C. Bechet J. M. Couderc L. J. Caubarrere I. Vernant J. C. (1989). Detection of HTLV-1 DNA by polymerase chain reaction in alveolar lymphocytes of patients with tropival spastic paraparesis. *J. Infect. Dis.* 160 162–163. 10.1093/infdis/160.1.162 2732511

[B8] Einsiedel L. Cassar O. Goeman E. Spelman T. Au V. Hatami S. (2014). Higher human T-lymphotropic virus type 1 subtype C proviral loads are associated with bronchiectasis in indigenous australians: results of a case-control study. *Open Forum Infect. Dis.* 1:ofu023. 10.1093/ofid/ofu023 25734096 PMC4324180

[B9] Einsiedel L. Pham H. Wilson K. Walley R. Turpin J. Bangham C. (2018). Human T-lymphotropic virus type 1c subtype proviral loads, chronic lung disease and survival in a prospective cohort of indigenous Australians. *PLoS Negl. Trop. Dis.* 12:e0006281. 10.1371/journal.pntd.0006281 29529032 PMC5874075

[B10] Falcão L. F. M. Falcão A. S. C. Sousa R. C. M. Vieira W. B. de Oliveira R. T. M. Normando V. M. F. (2017). CT Chest and pulmonary functional changes in patients with HTLV- associated myelopathy in the Eastern Brazilian amazon. *PLoS One* 12:e0186055. 10.1371/journal.pone.0186055 29095831 PMC5667869

[B11] Fukuoka J. Tominaga M. Ichikado K. Tanaka T. Ichiyasu H. Kohrogi H. (2013). Lung miliary micro-nodules in human T-cell leukemia virus type I carriers. *Pathol. Int.* 63 108–112. 10.1111/pin.12030 23464968

[B12] Grassi M. F. R. dos Santos N. P. Lirio M. Kritski A. L. Almeida M. C. C. Santana L. P. (2016). Tuberculosis incidence in a cohort of individuals infected with human T-lymphotropic vírus type 1 (HTLV-1) in Salvador, Brazil. *BMC Infect. Dis.* 16:491. 10.1186/s12879-016-1428-z 27643609 PMC5028977

[B13] Honarbakhsh S. Taylor G. P. (2015). High prevalence of bronchiectasis is linked to HTLV-1 - associated inflammatory disease. *BMC Infect. Dis.* 15:258. 10.1186/s12879-015-1002-0 26143070 PMC4491414

[B14] Kadota J. Mukae H. Fujii T. Seki M. Tomono K. Kohno S. (2004). Clinical similarities and differences between human T-cell lymphotropic virus type 1- associated bronchiolitis and diffuse panbronchiolitis. *Chest* 125 1239–1247. 10.1378/chest.125.4.1239 15078730

[B15] Kawabata T. Higashimoto I. Takashima H. Izumo S. Kubota R. (2012). Human T-lymphotropic virus type I (HTLV-I) Specific CD8+ Cells accumulate in the Lungs of patients infected with HTLV-I with pulmonary involvement. *J. Med. Virol.* 84 1120–1127. 10.1002/jmv.23307 22585731

[B16] Kikuchi T. Saijo Y. Sakai T. Abe T. Ohnuma K. Tezuka F. (1996). Human T-cell lymphotropic virus type I (HTLV-I) carrier with clinical manifestations characteristic of diffuse panbronchiolitis. *Intern. Med.* 35 305–309. 10.2169/internalmedicine.35.305 8739787

[B17] Kimura I. (1992). HABA (HTLV-1 associated bronchiolo-alveolar disorder). *Nihon Kyobu Shikkan Gakkai Zasshi* 30 787–795. 1630042

[B18] Kozlowski A. G. Carneiro M. A. S. Matos M. A. D. Teles S. A. Araujo Filho J. A. Otsuki K. (2014). Prevalence and genetic characterization of HTLV-1 and 2 dual infections in patients with pulmonary tuberculosis in Central-West Brazil. *Mem. Inst. Oswaldo Cruz* 109 118–121. 10.1590/0074-0276130230 24141955 PMC4005525

[B19] Maeda T. Babazono A. Nishi T. Yasui M. Matsuda S. Fushimi K. (2015). The impact of opportunistic infections on clinical outcome and healthcare resource uses for adult T cell leukaemia. *PLoS One* 10:e0135042. 10.1371/journal.pone.0135042 26274925 PMC4537272

[B20] Marinho J. Galvão-Castro B. Rodrigues L. C. Barreto M. L. (2005). Increased risk of tuberculosis with human T-lymphotropic virus-1 infection: a case control study. *J. Acquir. Immune Defic. Syndr.* 40 625–628. 10.1097/01.qai.0000174252.73516.7a 16284541

[B21] Matsuyama W. Kawabata M. Mizoguchi A. Iwami F. Wakimoto J. Osame M. (2003). Influence of human T lymphotrophic virus type I on cryptogenic fibrosing alveolitis - HTLV-I associated fibrosing alveolitis: proposal of a new clinical entity. *Clin. Exp. Immunol.* 133 397–403. 10.1046/j.1365-2249.2003.02240.x 12930367 PMC1808791

[B22] Mattos K. Melo A. Queiroz C. Publio L. Peçanha-Martins A. C. Freitas V. (1995). Pulmonary involvement in patients with HTLV-1associated myelopathy. *Arq. Neuropsiquiatr.* 53 766–770. 10.1590/S0004-282X1995000500009 8729770

[B23] Miyazato A. Kawakami K. Iwakura Y. Saito A. (2000). Chemokine synthesis and cellular inflammatory changes in lungs of mice bearing p40tax of human T-lymphotropic virus type 1. *Clin. Exp. Immunol.* 120 113–124. 10.1046/j.1365-2249.2000.01197.x 10759772 PMC1905621

[B24] Moreira E. D. Jr. Ribeiro T. T. Swanson P. Sampaio Filho C. Melo A. Brites C. (1993). Seroepidemology of human T-cell lymphotropic virus type I/II in northeastern Brazil. *J. Acquir. Immune Defic. Syndr.* 6 959–963. 8315579

[B25] Mori S. Mizoguchi A. Kawabata W. Fukunaga H. Usuku K. Maruyama I. (2005). Bronchoalveolar lymphocytosis correlates with human T lymphotropic vírus type I (HTLV-1) proviral DNA load in HTLV-1 carriers. *Thorax* 60 138–143. 10.1136/thx.2004.021667 15681503 PMC1747290

[B26] Murphy E. Ownby H. Smith J. Garraty G. Hutching S. Wu Y. (2003). Pulmonary function testing in HTLV-I and HTLV-II infected humans: a cohort study. *BMC Pulm. Med.* 3:1. 10.1186/1471-2466-3-1 12885299 PMC184441

[B27] Nakayama Y. Ishikawa C. Tamaki K. Senba M. Fujita J. Mori N. (2011). Interleukin-1 alpha produced by human T-cell leukaemia vírus type 1- infected T cells induces intercellular adhesion molecule-1 expression on lung epithelial cells. *J. Med. Microbiol.* 60 1750–1761. 10.1099/jmm.0.033456-0 21816944

[B28] Nakayama Y. Yamazato Y. Tamayose M. Atsumi E. Yara S. Higa F. (2013). Increased expression of HBZ and Foxp3 mRNA in bronchoalveolar lavage cells taken from human t-lymphotropic virus type 1-associated lung disorder patients. *Intern. Med.* 52 2599–2609. 10.2169/internalmedicine.52.0845 24292748

[B29] Normando V. M. Falcão L. F. M. Vieira W. Oliveira R. Santos M. Fuzii H. T. (2016). Changes in lung function in patients with human T cell lymphotropic virus (HTLV) associated myelopathy residents in the eastern Brazilian Amazon. *Eur. Respir. J.* 48 4442 10.1183/13993003.congress-2016.PA4442

[B30] Okada F. Ando Y. Yoshitake S. Yotsumoto S. Matsumoto S. Wakisaba M. (2006). Pulmonary CT Findings in 320 carriers of human T-lymphotropic virus type 1. *Radiology* 240 559–564. 10.1148/radiol.2402050886 16864677

[B31] Pedral-Sampaio D. B. Martins Netto E. Pedrosa C. Brites C. Duarte M. Harrington W. Jr. (1997). Co-infection of tuberculosis and HIV/HTLV retroviruses: frequency and prognosis among patients admitted in a Brazilian Hospital. *Braz. J. Infect. Dis.* 1 31–35. 11107236

[B32] Richardson J. H. Edwards J. Cruickshank J. K. Rudge P. Dalgleish A. G. (1990). In vivo cellular tropism of human T-cell leukemia virus type 1. *J. Virol.* 64 5682–5687. 1976827 10.1128/jvi.64.11.5682-5687.1990 PMC248630

[B33] Saito M. Bangham C.R. (2012) Immunopathogenesis of human T-cell leukemia virus type-1-associated myelopathy/tropical spastic paraparesis: recent perspectives. *Leuk. Res. Treatment* 2012:259045. 10.1155/2012/259045 23198155 PMC3505925

[B34] Seki M. Higashiyama Y. Mizokami A. Kadota I. Moriuchi R. Kohno S. (2000). Up-regulation of human T lymphotropic virus type 1 (HTLV-1) tax/rex mRNAS in infected lung tissues. *Clin. Exp. Immunol.* 120 488–498. 10.1046/j.1365-2249.2000.01237.x 10844528 PMC1905561

[B35] Sugimoto M. Nakashima H. Matsumoto M. Uyama E. Ando M. Araki S. (1989). Pulmonary involvement in patients with HTLV-1 associated myelopathy: increased soluble IL-2 receptors in bronchoalveolar lavage fluid. *Am. Rev. Respir. Dis.* 139 1329–1335. 10.1164/ajrccm/139.6.1329 2786358

[B36] Sugimoto M. Nakashima H. Watanabe S. Uyama E. Tanaka F. Ando M. (1987). T-lymphocyte alveolitis in HTLV-1 associated myelopathy. *Lancet* 2:1220 10.1016/S0140-6736(87)91362-6 2890850

[B37] Tateishi U. Nishihara H. Miyasaka K. (2001). HTLV-1-associated bronchopneumonopathy (HAB): CT-pathological correlation. *Clin. Radiol.* 56 664–666. 10.1053/crad.2001.0677 11467868

[B38] Taylor G. P. Matsuoka M. (2005). Natural history of adult T cell leukemia/lymphoma and approaches to therapy. *Oncogene* 24 6047–6057. 10.1038/sj.onc.1208979 16155611

[B39] Teruya H. Tomita M. Senba M. Ishikawa C. Tamayose M. Miyazato A. (2008). Human t-cell leukemia virus type I infects human lung epithelial cells and induces gene expression of cytokines, chemokines and cell adhesion molecules. *Retrovirology* 5 1–10. 10.1186/1742-4690-5-86 18808681 PMC2556696

[B40] Verdonck K. González E. Schrooten W. Vanham G. Gotuzzo E. (2008). HTLV-1 infection is associated with a history of active tuberculosis among family members of HTLV-1 infected patients in Peru. *Epidemiol. Infect.* 136 1076–1083. 10.1017/s0950268807009521 17892632 PMC2870904

[B41] Yamakawa H. Yoshida M. Yabe M. Ishikawa T. Takagi M. Tanoue S. (2015). Human T-cell lymphotropic virus Type-1 (HTLV-1)-associated bronchioloalveolar disorder presenting with mosaic perfusion. *Intern. Med.* 54 3039–3043. 10.2169/internalmedicine.54.4717 26631889

[B42] Yamamoto M. Matsuyama W. Odonakahara K. Watanabe M. Higashimoto I. Kawabata M. (2004). Influence of human T lymphotrophic virus type I on diffuse pan-bronchiolitis. *Clin. Exp. Immunol.* 136 513–520. 10.1111/j.1365-2249.2004.02485.x 15147354 PMC1809062

[B43] Yamashiro T. Kamiya H. Miyara T. Gibo S. Ogawa K. Akamine T. (2012). CT scans of the chest in carriers of human T-cell lymphotropic virus type 1: presence of interstitial pneumonia. *Acad. Radiol.* 19 952–957. 10.1016/j.acra.2012.03.020 22578413

[B44] Yamazato Y. Miyazato A. Kawakami K. Yara S. Kaneshima H. Saito A. (2003). High expression of p40tax and pro-inflammatory cytokines and chemokines in the lungs of human T-lymphotropic virus type 1- related bronchopulmonary disorders. *Chest* 124 2283–2292. 10.1378/chest.124.6.2283 14665512

